# Detection of parvovirus mRNAs as markers for viral activity in endomyocardial biopsy-based diagnosis of patients with unexplained heart failure

**DOI:** 10.1038/s41598-020-78597-4

**Published:** 2020-12-18

**Authors:** Heiko Pietsch, Felicitas Escher, Ganna Aleshcheva, Dirk Lassner, Claus-Thomas Bock, Heinz-Peter Schultheiss

**Affiliations:** 1 IKDT Institute of Cardiac Diagnostics and Therapy GmbH, Moltkestrasse 31, 12203 Berlin, Germany; 2 Department of Cardiology, Campus Rudolf Virchow, Charité-University Medicine Berlin, Berlin, Germany; 3 DZHK (German Centre for Cardiovascular Research), partner site Berlin, Germany

**Keywords:** Cardiomyopathies, Viral infection

## Abstract

*Erythroparvovirus* (B19V) genomes have been detected in various organs of infected individuals including endothelial cells of the heart muscle. However, the role of B19V as a causative pathogen of myocardial damage is still unknown. The majority of reports focus on the presence of viral DNA ignoring proof of viral RNAs as important markers for viral activity. During this study, we established (RT-) qPCR to characterize expression of B19V RNAs (NS1 and VP1/2) in endomyocardial biopsies (EMBs) of 576 patients with unexplained heart failure. 403/576 (70%) EMBs were positive for B19V DNA. B19V mRNAs NS1 and/or VP1/2, indicating viral activity, could be detected in 38.5% of B19V DNA positive samples using the newly established B19V RT-PCRs. 22.1% of samples were characterized by only NS1 mRNA detection while 6.0% revealed only VP1/2 mRNA expression. Detection of both intermediates was successful in 10.4% of samples. Applying the molecular testing, our study revealed that a high proportion (38.5%) of B19V DNA positive EMBs was characterized by viral transcriptional activity. Further prospective studies will evaluate relevance of viral transcription intermediates as a diagnostic marker to differentiate between latent B19V infection and clinically relevant transcriptionally active B19V-infection of the heart muscle.

## Introduction

Primate parvovirus (B19V), a non-enveloped single stranded linear DNA virus, belongs to the genus *Erythroparvovirus*. Infection with B19V is widespread in human population and leads to lifelong viral persistence^1^. As a consequence, B19V prevalence is increasing with age. Seroprevalence has been reported from 40 to 60% for young adults (< 20 years) to 78% at > 50 years of age^2^. B19V infection is usually asymptomatic or shows only mild symptoms. In contrast, B19V infection of immunosuppressed patients can cause transient aplastic crisis and persistent B19V infection developing as pure red cell aplasia and chronic anaemia^3^. During pregnancy B19V infection is associated with an increased risk for foetal loss or foetal hydrops^3^. B19Vgenomes have been detected in various organs such as heart, liver, kidney and skin, and different diseases such as rheumatoid arthritis and cutaneous T cell lymphomas^4–6^.

Myocarditis as an inflammatory disease of the myocardium may be idiopathic, infectious, or autoimmune. It may heal or lead to dilated cardiomyopathy (DCM) that is characterized by dilatation and impaired ventricular contraction. Myocarditis and DCM represent acute and chronic stages, whereas DCM is the third most common cause of heart failure^7^.

The classical viral pathogen associated with myocarditis or inflammatory dilated cardiomyopathy (DCMi) is Coxsackievirus B. However, B19V genomes are the most frequently detected viral genomes in endomyocardial biopsies (EMBs) of patients with suspected heart failure^8,9^. Since B19V DNA is frequently found in both, symptomatic and asymptomatic patients, the clinical relevance of B19V is still a matter of discussion^10–12^.

The 5.6 kb linear single stranded DNA genome contains two major open reading frames (ORFs) coding for the NS1 (non-structural protein) and VP1 and VP2 (capsid) proteins being flanked by inverted terminal repeat regions (ITRs) that are necessary for self-priming during viral genome replication. Two minor ORFs code for a 9 and 11 kDa protein of largely unknown function. Transcription activity is driven by a single promoter sequence (P6) while enhancer sequences upstream of the P6 promoter region recruit different cellular transcription factors^13^. From a single precursor (pre-) mRNA, 12 mature mRNAs are generated through alternative splicing that coordinates polyadenylation^14^. B19V infection induces a cell cycle arrest at late S phase and induces DNA damage response which will supply enzymes that facilitate B19V replication^15^. The expression of B19V transcription intermediates depends on splicing and polyadenylation efficiency and thus it depends on host cell factors. In semi-permissive cells, such as endothelial cells, transcripts are polyadenylated at polyadenylation site proximal (pA)p leading to an increased expression of NS1 intermediates. In permissive cells, however the blockade at (pA)p is overcome by replication of the viral genome in the late phase of infection and readthrough (pA)p leads to expression of VP1/2^16^.

Cardiomyocytes cannot be infected by B19V^8^. Host cell tropism of B19V is restricted to erythroid progenitor cells. Since endothelial cells of the myocardium express the primary erythrovirus receptor, the P-antigen, and co-receptors such as integrin α5β1 and KU80, infection with B19V of these semi-permissive cells leads to an incomplete viral replication cycle^17^. Due to the absence of certain host cell factors, B19V is not able to complete the viral replication cycle in endothelial cells, and thus no infectious progeny virions are produced^18^. Instead, infection of endothelial cells leads to endothelial dysfunction^18^. Several mechanisms may explain B19V mediated cytotoxicity, such as direct damage through the NS1 endonuclease domain or upregulation of pro-apoptotic signalling molecules e.g. TNFα or IL-6^19,20^. Furthermore, the VP1-unique region exerts phospholipase A2 activity that promotes inflammatory signalling and impairs endothelial function^21^. In addition, NS1 transactivates viral and host gene expression and is essential for viral replication due to its DNA nickase and helicase activity.

During this study, we established a molecular approach using newly generated RT-qPCRs to show viral activity by the detection and characterization of B19V NS1 and VP1/2 mRNAs in EMBs of patients with unexplained heart failure. Detection of viral RNA intermediates demonstrates viral activity and thus can be useful as a biomarker for B19V replication that allows to differentiate between latent and active B19V infection.

## Results

### Study subjects

EMBs of 576 patients with diagnosed unexplained heart failure were collected from 74 German clinical centres and sent to the Institute for Cardiac Diagnostics and Therapy GmbH for molecular analysis. The cohort comprised EMBs of 413 (71.7%) male and 163 (28.3%) female patients at a mean age of 53.6 ± 15.7 years with a mean left ventricular ejection fraction (LVEF) of 34.1% ± 15.7% at the date of hospitalization (Table 1). Routine analysis of B19V genome detection revealed that 403/576 (70%) of EMBs were positive for B19V DNA with a median viral load of 944.3 GE/µg ranging from 15 to 51,118 GE/µg (Table 1). However, determination of latent versus active B19V infection is pending and requires the analysis of viral transcription intermediates expression.

**Table 1:** Baseline characteristics of the study population (N = 576).

Characteristic	Value
Male, n (%)/female, n (%)	413 (71.7)/163 (28.3)
Age, years ± SD	53.6 ± 15.7
LVEF, % ± SD	34.1 ± 15.7
B19V genome detection, n/N (%)	403/576 (70)
B19 genomes viral load, median [GE/µg] (range)	944.3 (51,103)
Left/right ventricular catheterization, n (%)	303 (52.6%)/273 (47.4%)

### Establishment of B19V qPCR for B19V NS1 DNA and RNA detection

In order to assess the presence of B19V genomes and to assess viral activity of transcription in EMBs of patients with unexplained heart failure, PCRs targeting the B19V NS1 and VP1/2 regions were applied.

**Figure 1:**
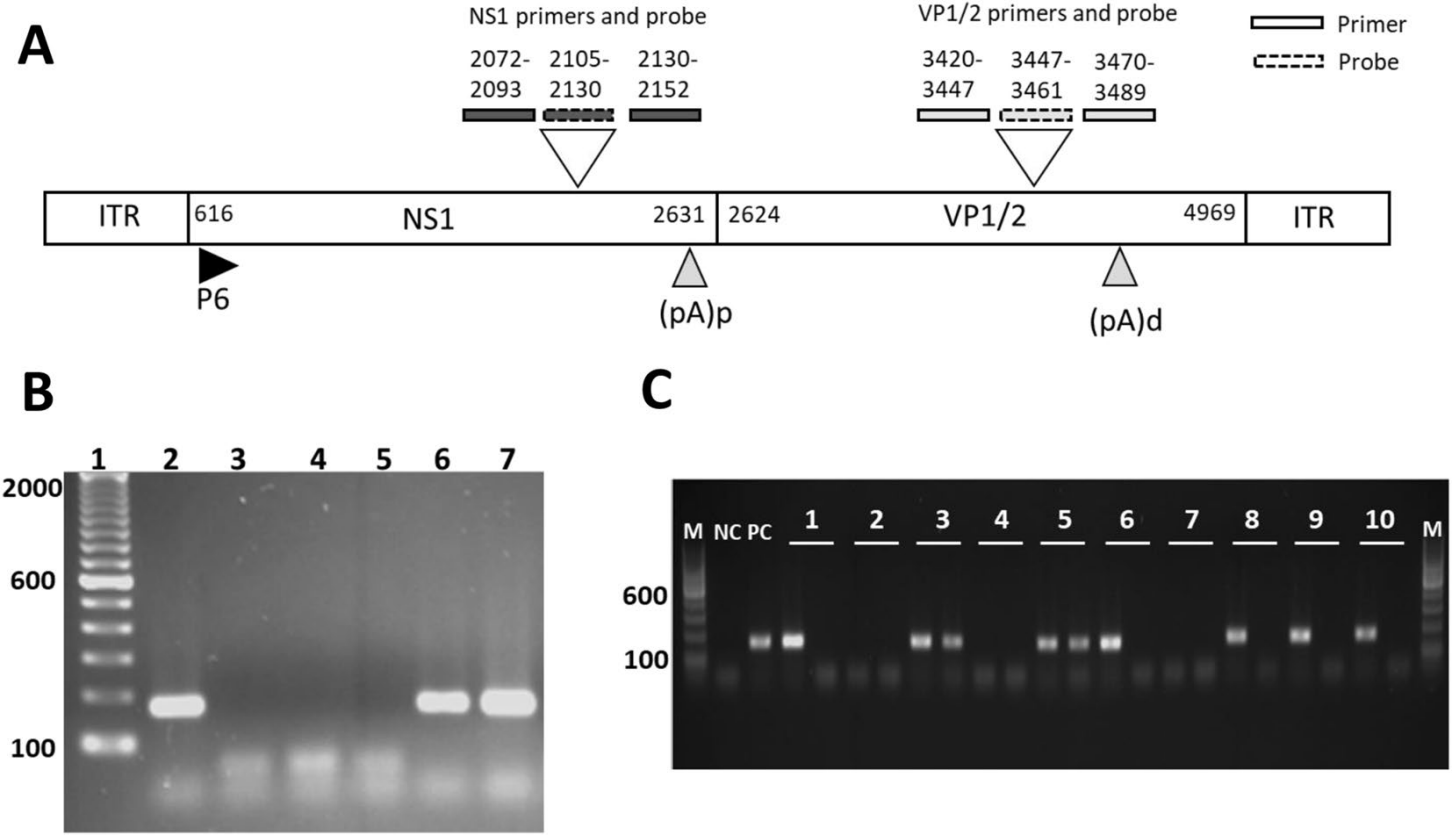
(**A**) Schematic description of the B19V genome organization and PCR design. Gene locus and primer and probe localization for NS1 and VP1/2 are indicated by numbers representing the nucleotide position. *ITR* inverted terminal repeat, *NS1 *non structural protein 1, *VP1/2 *capsid proteins, *P6 *P6 promotor, *(pA)p *polyadenylation site proximal, *(pA)d *polyadenylation site distal. (**B**) Representative agarose gel electrophoresis gel blot image of a B19V-VP1/2 DNA and RNA-positive EMB using VP1/2 specific nested-PCR. Amplicon length 173 bps. 1 = DNA-Marker 100 bps; 2 = positive control; 3 = negative control; 4 = PCR after DNA extraction and DNAse treatment; 5 = PCR after RNA extraction, RNAse treatment and RT-PCR; 6 = PCR after DNA extraction; 7 = PCR after RNA extraction and DNAse treatment and RT-PCR. Complete gel blot image of figure (**B**) was shown in Supplementary Fig. S1. (**C**) Representative agarose gel electrophoresis gel blot image of 10 EMB samples following VP1/2 specific nested PCR. DNA (first lane) and cDNA (second lane) of each EMB were analysed. Amplicon length 173 bps EMBs 1, 6, 8, 9 and 10 were tested positive for viral DNA and negative for viral RNA. EMBs 3 and 5 were positive for both, viral RNA and DNA. EMBs 2, 4 and 7 were virus negative without any viral DNA nor RNA being detectable. *M *100 bps marker, *NC *negative control, *PC *positive control.

Prototype genome sequences for the main B19V genotype 1 (J35; AY386330.1) and genotype 2 (LaLi; AY044266.2) were retrieved from NCBI GenBank. The sequences were globally aligned using MEGA Version X-Software (Version 10.0.5) by the implemented MUSCLE algorithm^22^. The obtained consensus sequence was then used as a reference sequence to design primers and probes for qPCR within the NS1 region (Fig. 1A). To account for differences in genotype-specific nucleotide sequences, degenerated nucleotides for primer design were used. The probes of the NS1 detection system were designed genotype-specific to allow for multiplex detection (Fig. 1A) (Table 2). Pairwise local alignment through Basic local alignment search tool (BLAST-algorithm) of each primer and probe sequence within the NCBI GenBank database confirmed the specificity of the designed sequences.

**Table 2:** Primer sequences used for detection of B19V.

Primer/probe name	Nucleotide sequence (5′–3′)	Nucleotide position^a^	1st, 2nd (RT)-nPCR/qPCR
NS1-FW	TCCCTGGAATWAATGCAGATGC	2072–2093	Sense B19V NS1 qPCR
NS1-RV	CACTGCTGCTGAYACTGGTGTCT	2130–2152	Antisense B19V NS1 qPCR
NS1-GT1-probe	6FAM-ACCTCCAAACCACCCCAATTGTCACA-TAMRA	2105–2130	Probe B19V NS1 qPCR
NS1-GT2-probe	VIC-ACCTCCAAACCGTCCCCATTGTCGCA-TAMRA	2105–2130	Probe B19V NS1 qPCR

^a^Nucleotide position according to reference sequence (AY386330.1).

### QPCR performance testing to detect B19V

The PCR was tested for residual cross contamination after DNA or RNA extraction. A representative image of agarose gel electrophoresis of a B19V DNA and RNA-positive EMB using VP1/2 specific nPCR primers was shown (Fig. 1B, Supplementary Fig. S1, Table 2). The amplicon was detectable after DNA extraction (Fig. 1B lane 6) and after RNA extraction, DNAse treatment and reverse transcription (Fig. 1B lane 7). No amplicon was detectable when DNAse treatment after DNA extraction was applied (Fig. 1B lane 5). After RNA extraction and RNAse treatment and reverse transcription PCR, no amplicon was detectable (Fig. 1B lane 4). The negative control (Fig. 1B lane 3) and the positive control with detectable amplicon at 173bps (Fig. 1B lane 2) were shown. Therefore, the DNAse treatment ensured that no DNA contamination is present in the sample after RNA extraction (Fig. 1B). A representative agarose gel of 10 EMBs following VP1 nPCR was shown (Fig. 1C). For each EMB, the viral DNA (first lane) and RNA (second lane) were shown (Fig. 1C). Whereas EMBs 1, 6, 8, 9, and 10 were positive for viral DNA, EMBs 3 and 5 were positive for both, viral RNA and DNA (Fig. 1C). EMBs 2, 4, and 7 were virus negative without any viral DNA nor RNA being detectable (Fig. 1C).

A serial dilution of the control plasmid (pParvovirus B19) ranging from 16,000 to 0.16 GE/µl demonstrated the performance of the NS1 qPCR (Supplementary Fig. S2). In each dilution stage, four replicates were measured and the mean value of corresponding Ct values ± SD were calculated (Supplementary Fig. S2). Successful NS1 detection was verified by VP1/2 PCR detection showing identical viral loads.

### B19V detection in EMBs of patients with unexplained heart failure

**Figure 2:**
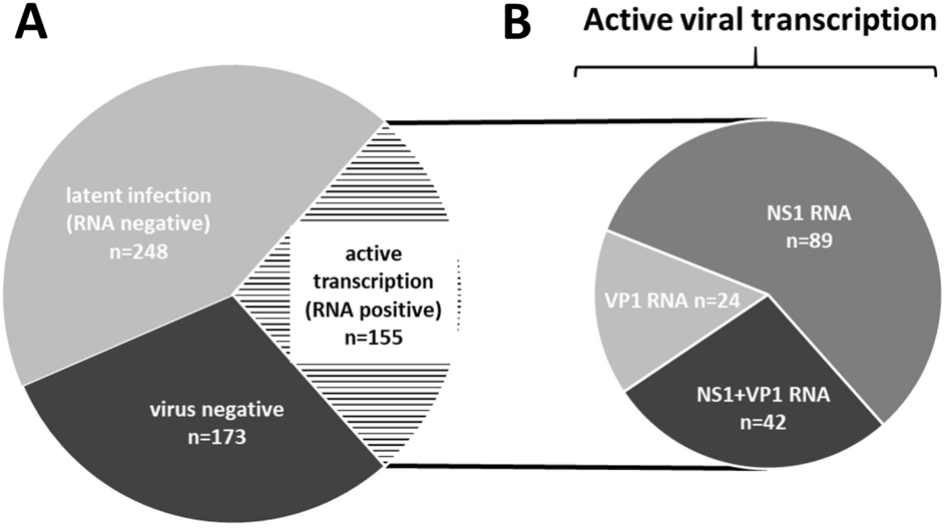
(**A**) B19V genome detection and detection of viral transcription activity in EMBs of patients with unexplained heart failure (N = 576). (**B**) The group composition of EMBs with detectable active viral transcription (VP1/2-RNA-, NS1-RNA and VP1/2 and NS1-RNA positive samples) was shown in detail. Numbers represent the amount of EMBs.

The majority of EMBs (70%; n = 403) of the total cohort (N = 576) showed B19V positive genome detection (DNA) using B19V-specific PCR (Fig. 2). B19V RNA detection using RT-qPCR assays revealed that 155/403 (38.5%) of analysed EMBs with detectable B19V DNA were positive for B19V RNA (Fig. 2A). 248/403 (61.5%) of these EMBs were characterized by a latent infection without any viral transcription intermediates being detectable (Fig. 2). Further analyses in terms of NS1 and VP1/2 RNA transcription determination in EMBs using NS1 and VP1/2-specific RT-qPCR showed that NS1 RNA could be detected in 89/403 EMBs (22.1%) positive for B19V genomes and expression of both, NS1 and VP1/2 RNA, in 42/403 samples (10.4%), respectively (Fig. 2B). However, in 24/403 samples (6%) only VP1/2-RNA intermediates were observed (Fig. 2B).

### Analysis of viral transcription and viral genome copy number

Detailed analysis on the occurrence of different forms of transcription intermediates demonstrated differences in viral transcription intermediates and viral genome copy number among these subgroups. These might therefore represent different entities of the disease.

Interindividual variation in DNA copy number (6545 ± 13,077 GE/µg) and RNA transcript number (4709 ± 11,713 GE/µg) were observed. Viral DNA load of NS1 correlated significantly with VP1/2-DNA load (p = 0.0148; Pearson r = 0.1228; r^2^ = 0.01509) and furthermore correlation of NS1- to VP1/2-RNA loads was highly significant (p ≤ 0.0001; Pearson r = 0.8817; r^2^ = 0.7775).

**Figure 3:**
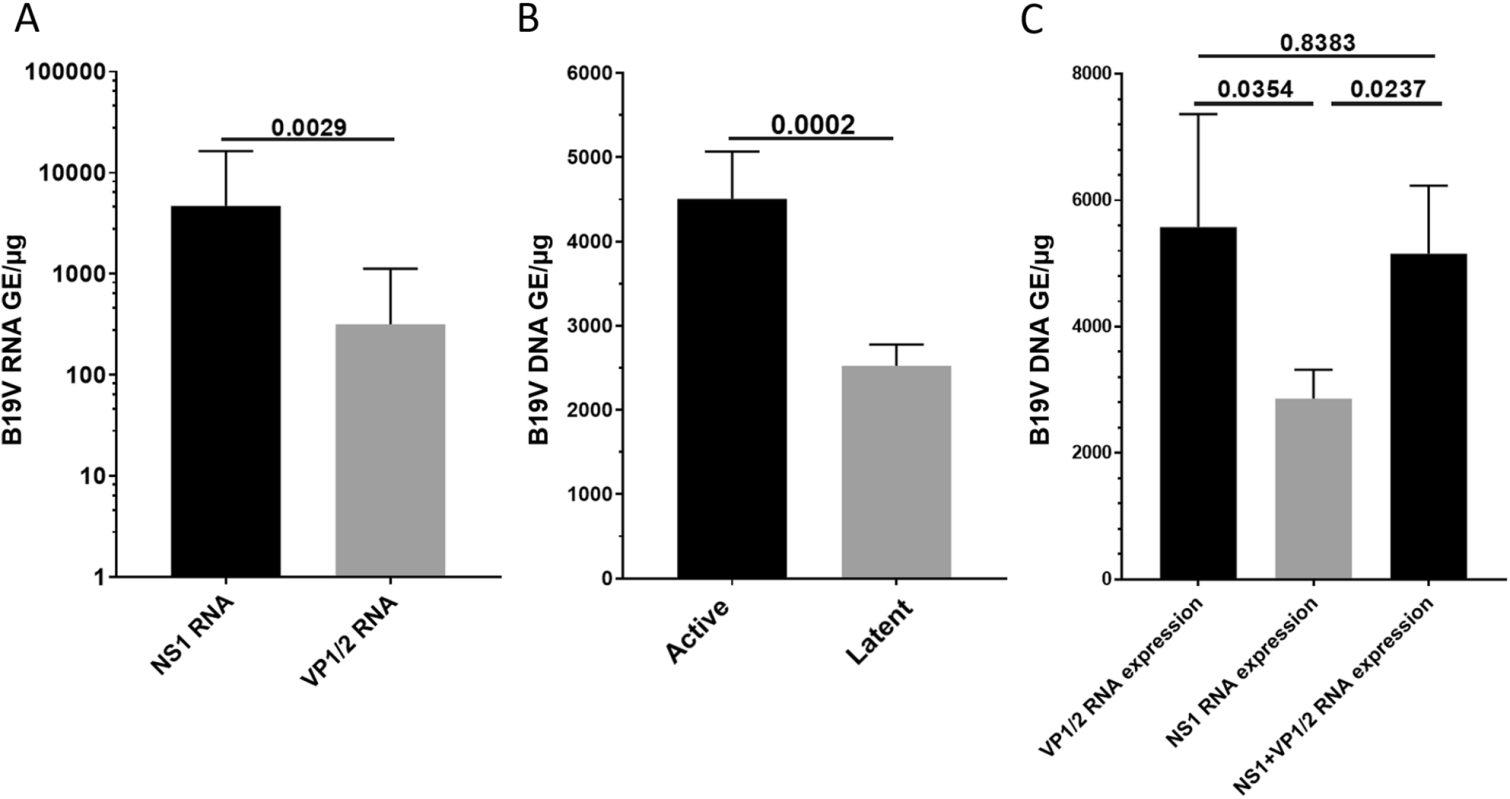
(**A**) The number of viral transcripts of NS1 compared to VP1/2. (**B**) Viral DNA loads in EMBs with active or latent infection. (**C**) Viral DNA load compared between EMBs with detectable VP1/2-RNA, NS1-RNA or NS1- and VP1/2-RNA expression (ANOVA p = 0.0427). Numbers above the bars represent p-values.

Expression level of NS1 transcripts (4709 ± 1023 GE/µg) differed significantly from the expression of VP1/2 transcripts (319.2 ± 100 GE/µg) (p = 0.0029) (Fig. 3A). EMBs of patients with active viral transcription being detectable presented with significantly increased DNA loads (4508 ± 562.6 GE/µg) when compared to EMBs of latently B19V infected patients (2526 ± 253.6 GE/µg) (p = 0.0002) (Fig. 3B). When both transcription intermediates (5156 ± 1076 GE/µg) or only VP1/2-RNA (5577 ± 1786 GE/µg) were detectable, the load of viral genomes was significantly increased compared to EMBs of patients that expressed solely NS1 RNA (2863 ± 454.2 GE/µg) (p = 0.0237; p = 0.0354) (Fig. 3C) Overall difference of viral load between these three groups was statistically significant (ANOVA p = 0.0427). Neither frequency of B19V detection nor viral load (p = 0.0763) or load of viral transcription intermediates (NS1: p = 0.9544; VP1: p = 0.3156; NS1 and VP1: p = 0.5190) were significantly different when left or right ventricular biopsy were compared.

166/248 (66.9%) of EMBs with latent viral infection exhibited a viral load of > 500 GE/µg. Significantly more EMBs with active viral transcription (124/155 (80%)) were characterized by a viral load of > 500 GE/µg (p = 0.0045). Baseline LVEF of patients affected by viral transcriptional activity (30.7 ± 13.1%) was significantly reduced when compared to virus free (35.5 ± 15.9%; p = 0.0254) or latently infected (34.7 ± 15.7%; p = 0.0432) patients, with no significant differences between virus free and latently infected patients (p = 0.7045).

## Discussion

B19V is a pathogen with broad clinical manifestations including myocarditis and chronic dilated cardiomyopathy (DCM). B19V genomes (B19V DNA) can be frequently detected in the routine molecular-pathology diagnostic in EMBs of unexplained heart failure, while with 36.7% B19V is the leading cardiotropic pathogen^12,23^. Since the first description of B19V as a cardiotropic pathogen in dogs and later on in humans, the clinical relevance of B19V infection of the heart muscle is a matter of discussion until to date^24^. However, since fulminant acute B19V infection in acute myocarditis can be causative for the heart disease is unquestionable, B19V infection in chronic myocarditis or DCM is still being discussed controversially^10–12^. Routine molecular diagnostic of cardiotropic pathogens is done using PCR techniques. However, B19V infection of the heart muscle is diagnosed only by B19V genome detection that cannot differentiate between latent and active B19V infection. B19V transcription intermediates such as B19V RNAs can therefore serve as markers of viral activity. Hence, we developed a new molecular approach to determine and quantify B19V RNAs in EMBs of patients with unexplained heart failure. Detection of B19V RNAs can differentiate between latent and active infection and therefore may serve as a marker for clinically relevant infection of the heart muscle.

In this regard, the present study demonstrated the successful detection of B19V genomes and the detection of B19V transcription intermediates (B19V RNAs) in a cohort of patients with unexplained heart failure. The newly established qPCRs targeting the NS1 and VP1/2 regions of the B19V genome demonstrated high specificity and sensitivity (Fig. 1, Supplementary Fig. S2). Among the EMBs of 576 consecutive patients suffering from unexplained heart failure who underwent first diagnostic endomyocardial biopsy, B19V genomes could be identified in 70% of all EMBs analysed. Furthermore, viral activity was detected in 26.9% of all EMBs analysed by detection of viral mRNA transcripts using the newly generated B19V-specific RT-qPCR.

The finding of 70% of B19V positivity in EMBs of the total cohort is in line with observations of a previous report^25^. A previous meta-analysis revealed that the overall detection rate of B19V genomes by PCR in different tissue types was 44.8% as summarized from 18 studies^4^. Since B19V prevalence increases with age, the rate of persistently infected individuals will be affected by the mean age of the sample population. A seroprevalence of 78% has been reported for individuals > 50 years of age that corresponds well to the data of our study population (mean age 53.6 ± 15.7 years)^2^. However, the high detection rate of active viral transcription by RT-qPCR (38.5% of all B19V positive EMBs) has not been reported before. We could also show that it was crucial to search for both replication intermediates in EMBs, the NS1 and VP1/2 viral RNAs. For that reason, our data demonstrated that for NS1 and VP1/2 RNAs in 57.4% and 15.5%, respectively, of B19V-positive EMBs an active viral transcription will remain undetected if only VP1/2 or NS1 intermediates are detected during molecular diagnostics using our newly established molecular approach. Left or right ventricular catheterization did not bias detection rate or viral load of B19V. Results were in accordance with a previous study reporting no significant difference in B19V genome detection rate when left and right ventricular EMBs were analysed in parallel^26^.

Previous studies reported that persistence of B19V DNA in the heart muscle is a common finding in EMB-based analysis and could not be correlated to clinical symptoms^10,11,27,28^. In contrast, other reports revealed that B19V infection of the heart muscle may lead to diastolic dysfunction and was associated with DCMi^8,29^. Furthermore, progressive cardiac dysfunction in the course of B19V infection has been linked to viral persistence^30^. To date only few studies investigated the pathogenic effects of B19V with respect to viral activity^31^. EMBs from healthy donors were not available for ethical reasons in the present study and comparison of B19V transcriptional activity between control and cardiac patients is pending and will be accomplished in future studies. The high number of B19V genome detection both, in healthy controls and in EMBs from patients with myocarditis or dilated cardiomyopathy, may be due to the fact that up to 70% of healthy individuals suffered from a past B19V infection depending on progressing age^1,28^. However, viral genomes in all tissues including the heart muscle will not be cleared following infection but remain generally as a latent infection^1^. The expression of viral transcription intermediates represents a distinct entity of the disease with clinical consequences for the patient. From a cohort of 416 B19V DNA positive cardiac patients, in only a small fraction (15.9%) active viral transcription was detectable^31^. Bock et al. reported that viral transcripts were only detectable in patients with myocardial inflammation and were absent in persistently infected patients without viral transcription^12^. Viral transcription might be associated with local increase in inflammatory signalling while triggering of signalling cascades may lead to increased expression of IL-6 or TNF-α^19,20,32^. Since detection of B19V genomes is a common finding in various diseases, the viral activity as measured by transcription of viral mRNAs, will be of potential diagnostic relevance. We hypothesize that reduced LVEF at baseline of patients presenting with viral transcription activity compared to latently infected or virus free patients might be associated with pathogenic effects of B19V transcription activity. However, this finding must be confirmed by further prospective studies also assessing long-term effects of B19V in the myocardium. Variation in viral loads and viral RNA expression among samples were observed which may be attributed to different phases of the B19V replication cycle. As we could demonstrate here, the highest amount of viral DNA was found in those samples when both viral RNAs, NS1 and VP1/2, or only VP1/2-RNA were detectable (Fig. 3C). This finding is in line with few studies that used both, capsid (VP1/2) and non-structural protein (NS1) specific sequences to detect B19V genomes by PCR, while discrepancies in the detection rate were reported^4^. Furthermore, our analysis revealed that EMBs of patients with active transcription demonstrated a significantly increased viral load (Fig. 3B). This result is in agreement with a previous study by Kuehl et al. showing that the expression of viral VP1/2 intermediates lead to significant increase in viral genomes while a correlation between the number of viral RNA transcripts and the viral genome copy number was observed^31^. Our data are furthermore supported by in vitro studies showing that replication of B19V proceeds in different phases and viral DNA and RNA concentrations may vary during these phases. The early phase of replication is characterized by alternative splicing and internal polyadenylation of pre-mRNA and leads to expression of NS1^33^. Whereas the replication of the viral genome and the formation of double-stranded viral DNA initiates the shift of gene expression towards an increase in VP1/2 during the late phase of infection^33^.

Notably, in vitro studies revealed that in semi-permissive cells such as endothelial cells the replication cycle of B19V is not able to complete due to absence of cellular host factors that drive the viral genome replication. Parvoviral replication is limited to erythroid progenitor cells in vivo and to certain cell types in vitro. Therefore, infection of endothelial cells does not lead to a productive infection. As a consequence, VP1/2 expression is low and NS1 is expressed at a higher rate^34^. Accumulation of NS1 may trigger the hosts’ immune response leading to auto-immunity that could be responsible for cardiac damage and clinical symptoms. Endothelial dysfunction then leads to secondary necrosis of myocardial cells^29^. Furthermore, NS1 exhibits cytotoxicity through mitochondria-mediated reactive oxygen species accumulation and apoptosis. For porcine parvovirus, it has been reported that NS1 expression leads to a downregulation of antiapoptotic molecules Bcl-2 and Mcl-1 and enhanced expression of proapoptotic molecules Bax, P21, and P53^35^. In addition, a direct damage of B19V in vivo might be possible through its NS1 endonuclease function^36^.

Only recently, novel antiviral treatment options against B19V have been discussed. In this regard, telbivudine, a nucleoside analogue used for Hepatitis B-virus therapy, seemed to be promising^37^. In order to monitor B19V replication activity under antiviral treatment, e.g. with telbivudine, viral RNA detection will be a pivotal diagnostic method.

## Study limitation

Limitations typical for retrospective cohort studies apply to our analyses. These include, among other factors, a lack of extended clinical data for all of the patients covered in this study. Prospective studies must investigate long-term effects of B19V activity in the myocardium. EMBs from healthy donors were not available for ethical reasons in the present study and comparison of B19V transcriptional activity of control and cardiac patients is pending and must be addressed in future studies. Limitation in sample material available of each patient did not allow for intensive investigation, such as virus-host interactions.

## Conclusion

In this study, we developed new molecular approach for B19V genome detection in order to discriminate a latent B19V infection from a B19V infection that is characterized by viral transcriptional activity in EMBs of patients suffering from unexplained heart failure. Retrospective analysis of 576 EMB samples demonstrated the feasibility of the method in a clinical setting of molecular diagnostics. The detection of B19V RNA replication intermediates can serve as a novel biomarker to differentiate between clinically relevant and non-relevant B19V infection.

## Methods

### Acquisition of endomyocardial biopsy samples

EMBs of 576 consecutive patients with clinical evidence of symptomatic heart failure of unknown cause after invasive exclusion of coronary artery disease by left heart catheterization (e.g., acute cardiac decompensation and suspected acute myocarditis) were collected from 74 German clinical centres and were sent to the Institute for Cardiac Diagnostics and Therapy GmbH for molecular diagnostics. All patients enrolled were catheterized for EMB during the period of 03/16/2011 to 08/25/2008 (median: 11/16/09) in the clinical centres. Since consecutive patients were investigated in the present study, the cohort represents a cross-section of cardiac patients catheterized during acute or stable phase. Patients suffering from a severe course of disease, such as fulminant acute forms of heart muscle disease, were excluded. The cohort comprised EMBs from 413 (71.7%) male and 163 (28.3%) female patients at a mean age of 53.6 ± 15.7 years with a mean left ventricular ejection fraction of 34.1% ± 15.7% at the date of hospitalization. All samples were retrospectively and anonymously tested for B19V. The extended diagnostic approach of the present study was approved by and performed within European Research Area Network on Cardiovascular Diseases (ERA-CVD; JTC2016-40-158).

### Sample preparation

Immediately after taking EMBs at the hospitals, samples were transferred to RNAlater solution (Thermo Fisher Scientific, Waltham, MA, USA) stabilizing the nucleic acids of the EMBs^38^. DNA from two to three EMBs was extracted by Puregene Core Kit A (Qiagen, Hilden, Germany) according to manufacturer’s instructions^31,38,39^. Total RNA was isolated using TRIzol Reagent (Thermo Fisher Scientific, Waltham, MA, USA), treated with DNAse (PeqLab, Erlangen, Germany) to remove any traces of DNA, and reverse-transcribed to cDNA with High-Capacity cDNA Reverse Transcription Kit (Thermo Fisher Scientific, Waltham, MA, USA) using random hexamer primers according to the manufacturers protocol (Thermo Fisher Scientific, Waltham, MA, USA)^31,38–40^. Following cDNA synthesis or DNA extraction, samples were stored at − 80 °C until further evaluation^40^. Nucleic acid concentration was measured by PCR-based Quantifiler Human DNA Quantification Kit (Thermo Fisher Scientific, Waltham, MA, USA) according to the manufacturer´s instructions^38,40^.

### Molecular diagnostics

#### VP1/2-detection

Nested polymerase chain reaction (nPCR) and quantitative real time PCR (qPCR) targeting the VP1/2 region of B19V were applied to detect B19V genomes (DNA) and VP1/2 mRNA as described previously^12,17^.

#### NS1-DNA detection

For the newly developed NS1-qPCR, 4 µl of extracted sample DNA and primers NS1-FW and NS1-RV and probes NS1-GT1-probe and NS1-GT2-probe were used. The PCR reaction was carried out in a 96-well microtiter plate format (Applied Biosystems, USA) according to the manufacturer’s instructions using TaqMan Universal PCR Master Mix (Applied Biosystems, USA) on a QuantStudio 12 K Flex Real-Time PCR System (Applied Biosystems, USA) (Table 2). Serial dilutions of a plasmid (pParvovirus B19) containing the NS1 sequence (3.5 to 3.5 × 10^4^ GE/µl) (GenExpress, Berlin, Germany) were simultaneously amplified for quantification and standardization as described previously^41^ (Table 2). Viral load was calculated by ratio of viral genome copy number to amount of total DNA extracted and was given as viral genome equivalents/µg genomic DNA (GE/µg). For qPCR of NS1 the following reaction conditions were used: initial denaturation for 10′ at 95 °C, followed by 40 cycles of denaturation for 15″ at 95 °C and a combined annealing and extension step for 60″ at 60 °C.

#### NS1-RNA detection

The newly developed NS1-qPCR was carried out in a 96-well microtiter plate format (Applied Biosystems, USA) according to the manufacturer’s instructions using TaqMan Universal PCR Master Mix (Applied Biosystems, USA) using 4 µl of extracted sample cDNA and primers NS1-FW and NS1-RV and probes NS1-GT1-probe and NS1-GT2-probe on a QuantStudio 12 K Flex Real-Time PCR System (Applied Biosystems, USA) (Table 2). Serial dilutions of a plasmid (pParvovirus B19) containing the NS1 sequence (3.5 to 3.5 × 10^4^ GE/µl) (GenExpress, Berlin, Germany) were simultaneously amplified for quantification and standardization as described previously^41^ (Table 2). Copy numbers of viral RNA were normalized by quantification of isolated total mRNA measured as expression of the house-keeping gene HPRT. Expression of HPRT was measured using predesigned primers and probe (TaqMan gene expression assay; Hs99999909_m1) (Applied Biosystems, Germany) and therefore also served as an internal quality control for extraction efficiency or possible sample degradation. Serial dilutions (25–2 ng/µl) of reverse transcribed Total RNA Control (Human) (Applied Biosystems, Germany) were used to quantify HPRT expression. The PCR reaction was conducted according to the manufacturer’s instructions using TaqMan Universal PCR Master Mix (Applied Biosystems, USA). For qPCR of NS1 the following reaction conditions were used: initial denaturation for 10′ at 95 °C, followed by 40 cycles of denaturation for 15″ at 95 °C and a combined annealing and extension step for 60″ at 60 °C.

### Statistical analysis

Results for quantitative analysis are given as mean value ± SD (standard deviation). Fisher’s exact test and Chi-square test were used to compare frequency distribution of dichotomic variables among two or more groups. To compare continuous variables between two groups, parametric unpaired Student’s *t*-test or non-parametric Mann–Whitney U test was used in the case of not normally distributed data, respectively. To test for statistical significance between more than two groups, ANOVA or Kruskal–Wallis test was used. For correlation analysis, Pearson correlation coefficient (r^2^) was assessed. P-values below 0.05 were considered to indicate statistical significance. All statistical analyses were performed using GraphPad Prism 7.04 software (GraphPad Software Inc., La Jolla, CA, USA). All graphics were created using GraphPad Prism 7.04 software (GraphPad Software Inc., La Jolla, CA, USA).

### Ethical approval

Extended routine diagnostic has been applied for and was approved within the European Research Area Network on Cardiovascular Diseases (ERA-CVD; JTC2016-40-158). The study conformed to the principles outlined in the Declaration of Helsinki. Patients’ data were anonymized for analyses. All experimental methods applied during the study were approved by the ethics committee of Charité-Universitätsmedizin Berlin, Germany, (Ethikkommission, Ethikausschuss 4 am Campus Benjamin Franklin, Charitéplatz 1, 10117 Berlin) (Ethikvotum Berlin 225-07) within the SFB Transregio 19 (Deutsche Forschungsgemeinschaft (DFG), project number 5486135).

### Informed consent

Informed written consent was obtained from each study patient.

## Supplementary Information

Supplementary Information.
