# Supplementary material for: Detection of parvovirus mRNAs as markers for viral activity in endomyocardial biopsy-based diagnosis of patients with unexplained heart failure
Source: Sci Rep. 2020 Dec 18;10:22354. doi: 10.1038/s41598-020-78597-4 (PMC7749156; doi:10.1038/s41598-020-78597-4)
Supplement: Supplementary file 1 — Supplementary Information. [file 41598_2020_78597_MOESM1_ESM.pdf]

Detection of parvovirus mRNAs as markers for viral activity in endomyocardial biopsy-based diagnosis of patients with unexplained heart failure

Short Title: B19V RNA detection as diagnostic marker for viral activity

Pietsch H[1,2,3], Escher F[1,2,3], Aleshcheva G[1], Lassner D[1], Bock CT[1], Schultheiss HP[1,*]

[1]Institute of Cardiac Diagnostics and Therapy GmbH, Berlin, Germany

[2]Department of Cardiology, Campus Rudolf Virchow, Charité - University Medicine Berlin, Berlin, Germany

[3]DZHK (German Centre for Cardiovascular Research), partner site Berlin, Germany

[*]Corresponding author: Heinz-Peter Schultheiss, M.D.

IKDT Institute of Cardiac Diagnostics and Therapy

Moltkestrasse 31, D-12203 Berlin, Germany

Email: heinz-peter.schultheiss@ikdt.de

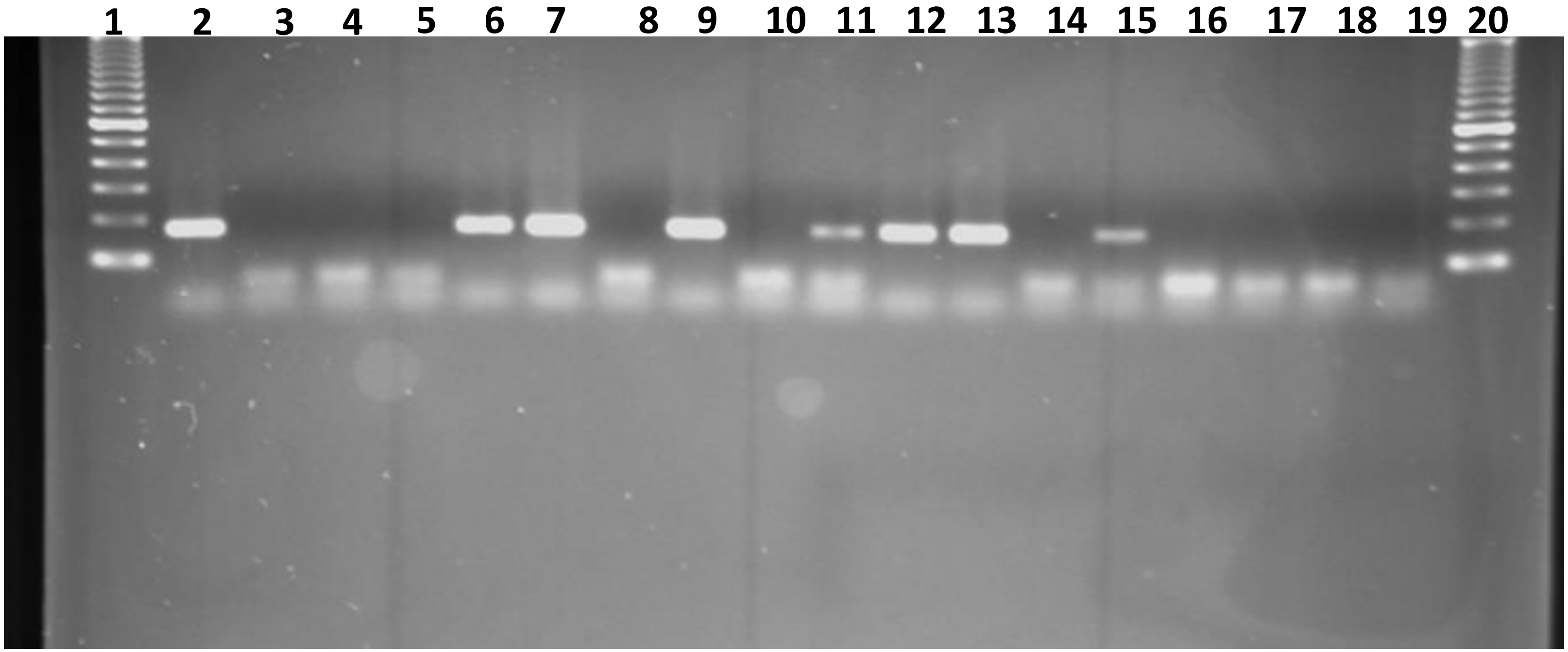

Supplementary Figure S1: Complete gel blot image of cropped gel blot image Fig. 1 (B)
Representative agarose gel electrophoresis gel blot image of a B19V-VP1/2 DNA and RNA-positive EMB using VP1/2 specific nested-PCR (lane 1-7) and . Amplicon length 173 bps. 1 = DNA-Marker 100 bps; 2 = positive control; 3 = negative control; 4 = PCR after DNA extraction and DNAse treatment; 5 = PCR after RNA extraction, RNAse treatment and RT-PCR; 6 = PCR after DNA extraction; 7 = PCR after RNA extraction and DNAse treatment and RT-PCR; 8 = EMB 1, negative for viral RNA; 9 = EMB 1 positive for viral DNA; 10 = EMB 2, negative for viral RNA; 11 = EMB 2 positive for viral DNA; 12 = EMB 3 positive for viral RNA; 13 = EMB 3 positive for viral DNA; 14 = EMB 4 negative for viral RNA; 15 = EMB 4 positive for viral DNA; 16 = EMB 5, negative for viral RNA; 17 = EMB 5, negative for viral DNA; 18 = EMB 6, negative for viral RNA; 19 = EMB 6, negative for viral DNA; 20 = DNA-Marker 100 bps

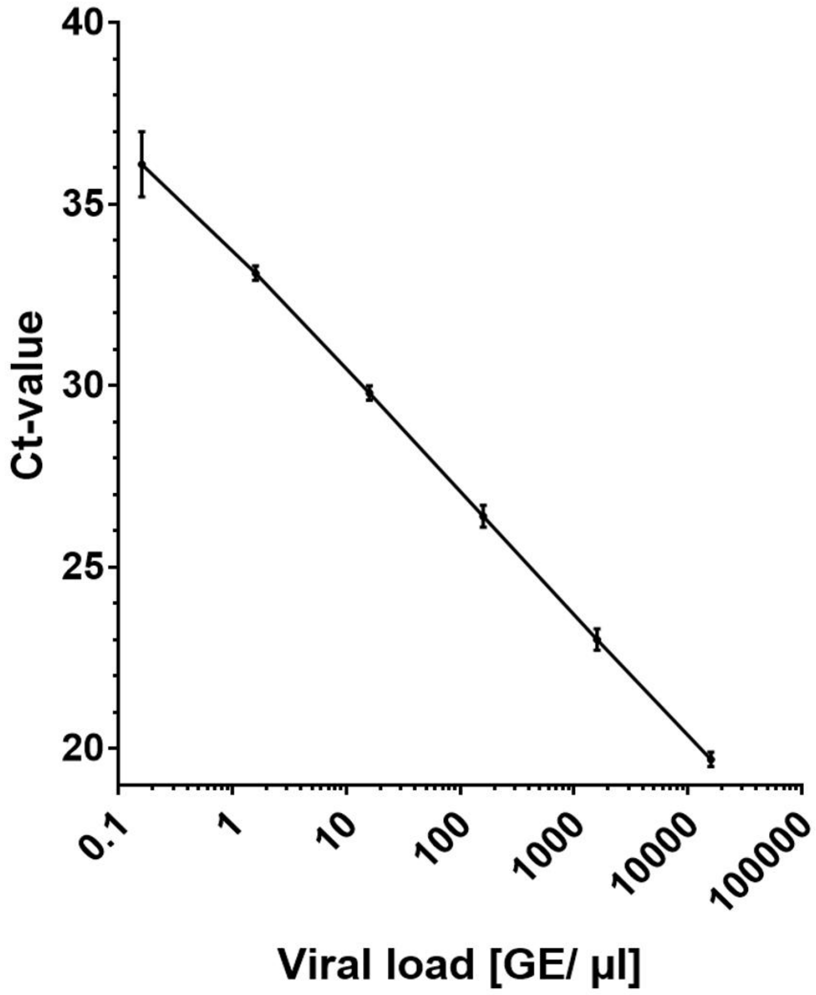

Supplementary Figure S2: Performance of NS1 qPCR amplification of pParvovirus B19 control plasmid
Serial dilution of control plasmid (pParvovirus B19) ranging from 0.16 to 16,000 copies/ µl to demonstrate performance of NS1 qPCR. Four replicates were measured in each dilution stage and mean Ct-values and SD are depicted
